# Clinicopathological characteristics and prognosis of signet ring cell carcinoma of the gallbladder

**DOI:** 10.1186/s12876-021-01831-4

**Published:** 2021-06-06

**Authors:** Shijie Wang, Jiayi Li, Jun You, Yanming Zhou

**Affiliations:** Department of Hepatobiliary and Pancreatovascular Surgery, The First Affiliated Hospital of Xiamen University, Xiamen, 361003 China

**Keywords:** Signet ring cell carcinoma, Gallbladder, Surgery, Prognosis

## Abstract

**Background:**

Signet ring cell carcinoma (SRC) is a rare histological subtype of gallbladder adenocarcinoma. The current study evaluates the clinicopathologic features and prognosis of SRC.

**Methods:**

Patients with adenocarcinoma of the gallbladder were identified in the Surveillance, Epidemiology, and End Results database from 1973 to 2016. Overall survival (OS) and cancer-specific survival (CSS) of patients who had SRC were compared with those of patients who had non-SRC using Cox regression and propensity score methods.

**Results:**

Of 22,781 gallbladder adenocarcinomas retrieved, 377 (1.7%) were SRC and the other 22,404 were non-SRC. SRC was more significantly associated with older age, female gender, poor differentiation, advanced tumor stage, lymph node metastasis, distant metastasis, and advanced AJCC stage. The 5-year OS and CSS in the SRC group were 7.2 and 6.5%, respectively, both of which were significantly worse than the 13.2 and 13.3% seen in the SRC group (*P* = 0.002 and *P* = 0.012, respectively). This survival disadvantage persisted in multivariable analyses [hazard ratio (HR) = 1.256, *P* = 0.021 and HR = 1.211, *P* = 0.036] and after propensity score matching (OS: HR = 1.341, *P* = 0.012 and CSS: HR = 1.625, *P* = 0.005). Surgery in combination with chemotherapy improved OS of gallbladder SRC patients compared with surgery alone (HR = 0.726, *P* = 0.036) or chemotherapy alone (HR = 0.433, *P* < 0.001).

**Conclusion:**

Patients with SRC of the gallbladder have distinct clinicopathological features with poor prognosis. Surgery in combination with chemotherapy can improve survival.

## Background

Signet ring cell carcinoma (SRC) is an adenocarcinoma in which more than 50% of the tumor consists of isolated or small groups of malignant cells containing intracytoplasmic mucins [1]. More than 96% SRCs arise in the stomach, accounting for 11–37% of all gastric cancers [2–5]. SRC of the gallbladder is extremely rare, and little is known about the clinicopathological characteristics, prognosis, and optimal treatment. We sought to address this issue through the Surveillance, Epidemiology, and End Results (SEER) database, a large population-based cancer registry.

## Methods

### Data source and study cohort

The adenocarcinoma of the gallbladder part in the SEER database diagnosed from 1973 to 2016 was the source of present analysis. The diagnosis of SRC and non-SRC was according to the third edition of the International Classification of Disease for Oncology (ICD-O) code 8490 and 8140 respectively. Patients with no follow-up or vital status information were excluded. Meanwhile, patients with non-primary tumors and no pathologic diagnosis were excluded. The American Joint Committee on Cancer (AJCC) staging manual (7th edition) was applied in this study. The main outcomes were overall survival (OS) and cancer-specific survival (CSS).

### Statistical analysis

Categorical variables were compared using a Pearson χ^2^ tests or Fisher exact test. The Kaplan–Meier method was used to calculate survival curves, and the log-rank test was used to identify statistically significant covariates associated with survival in univariate analysis. To identify independent risk factors of survival, multivariate Cox proportional hazard models were applied. In addition, a propensity score matching (PSM) analysis was performed to adjust for all potential baseline confounding variables in the two groups. A *P* value less than 0.05 was considered statistically significant. Data was analyzed using SPSS (version 24.0; SPSS, Inc., Chicago, IL).

## Results

**Fig. 1:**
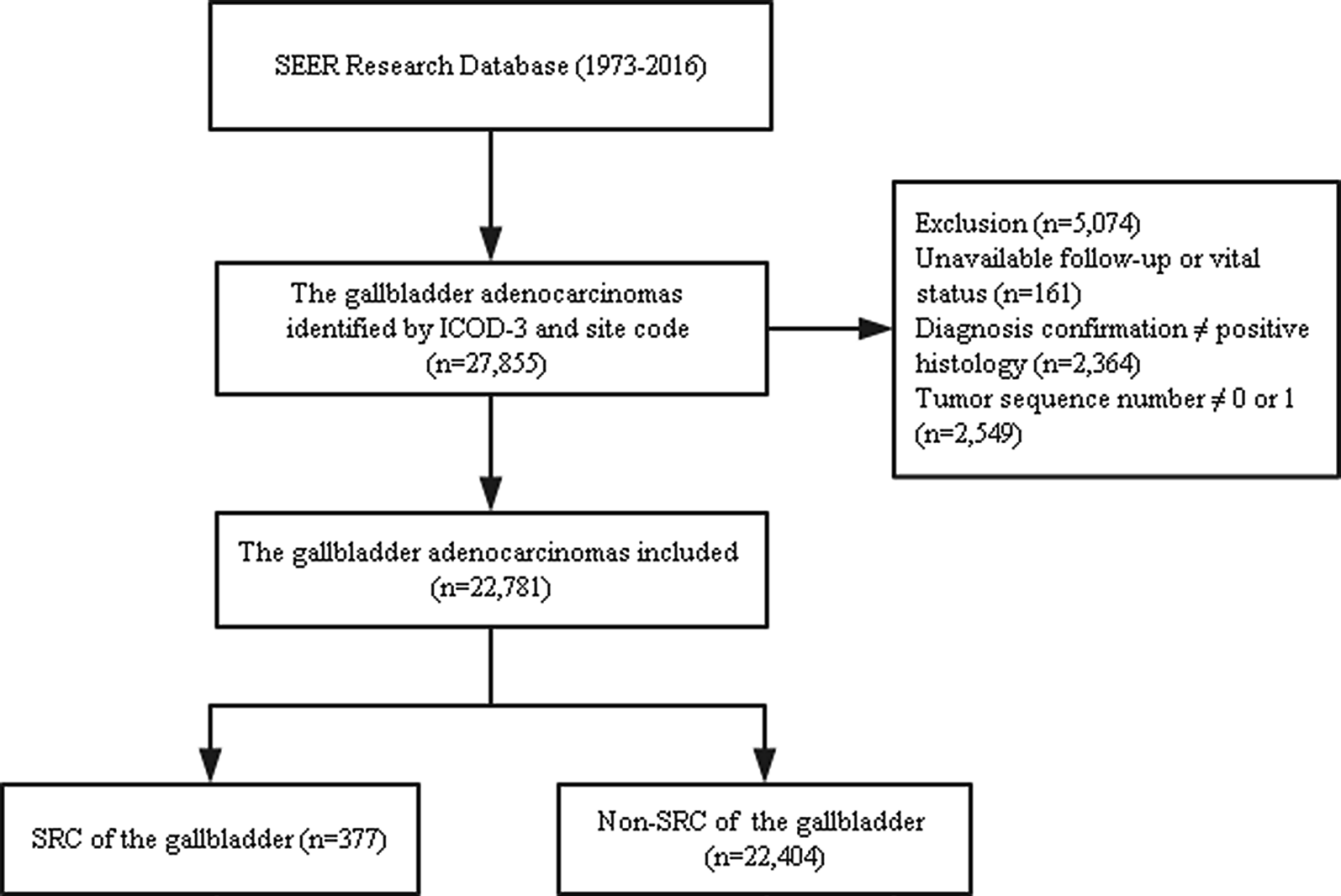
Flow diagram of patient selection. SRC, signet ring cell carcinoma; non-SRC, non-signet ring cell carcinoma

Of the 22,781 gallbladder asenocarcinomas included in this study, 377 (1.7%) were SRC and the other 22,404 were non-SRC (Fig. 1). The median follow-up duration was 6 months. At the end of the follow-up period, 3050 patients (13.4%) were alive, 13,890 patients (61.0%) died from cancer, and 5841 (25.6%) patients died of other causes.

The clinicopathological characteristics of the patients are listed in Table 1. SRC was more significantly associated with older age, female gender, poor differentiation, advanced tumor stage, lymph node metastasis, distant metastasis, and advanced AJCC stage. Regarding treatment, more SRC patients received surgery, radiotherapy and chemotherapy than non-SRC patients.

**Table 1:** Baseline demographic and clinicopathological characteristics of patients with SRC *vs.* non-SRC

Parameters	SRC (n = 377)	Non-SRC (n = 22,404)	*P* Value
*Age, years*			
< 60	83 (22.0%)	6954 (31.0%)	< 0.001
≥ 60	294 (78.0%)	15,450 (69.0%)	
*Sex*			
Male	88 (23.3%)	6625 (29.6%)	0.009
Female	289 (76.7%)	15,779 (70.4%)	
*Race*			
White	291 (77.2%)	17,781 (79.4%)	0.535
Black	44 (11.7%)	2264 (10.1%)	
Other	42 (11.1%)	2359 (10.5%)	
*Clinical T-stage*			
T1–T2	87 (23.1%)	4750 (21.2%)	< 0.001
T3–T4	121 (32.1%)	5248 (23.4%)	
Unknown	169 (44.8%)	12,406 (55.4%)	
*Lymph node metastasis*			
No	115 (30.5%)	6802 (30.4%)	< 0.001
Yes	91 (24.1%)	3158 (14.1%)	
Unknown	171 (45.4%)	12,444 (55.5%)	
*Distant metastasis*			
No	133 (35.3%)	6693 (29.9%)	< 0.001
Yes	93 (24.7%)	4445 (19.8%)	
Unknown	151 (40.1%)	11,266 (50.3%)	
*AJCC stage*			
I–II	118 (31.3%)	5811 (25.9%)	< 0.001
III–IV	105 (27.9%)	5054 (22.6%)	
Unknown	154 (40.8%)	11,539 (51.5%)	
*Histologic grade*			
Well-moderate	23 (6.1%)	8629 (38.5%)	< 0.001
Poor-undifferentiated	269 (71.4%)	6588 (29.4%)	
Unknown	85 (22.5%)	7187 (32.1%)	
*Surgery*			
Yes	246 (65.3%)	7138 (31.9%)	< 0.001
No	66 (17.5%)	3556 (15.9%)	
Unknown	65 (17.2%)	11,710 (52.3%)	
*Radiotherapy*			
Yes	52 (13.8%)	2354 (10.5%)	0.04
No	325 (86.2%)	20,050 (89.5%)	
*Chemotherapy*			
Yes	125 (33.2%)	6006 (26.8%)	0.006
No	252 (66.8%)	16,398 (73.2%)	
*Year of diagnosis*			
1975–2009	249 (66.0%)	15,676 (70.0%)	0.100
2010–2016	128 (34.0%)	6728 (30.0%)	
*Marital status*			
Married	183 (48.5%)	11,854 (52.9%)	0.092
Unmarried	194 (51.5%)	10,550 (47.1%)	

*SRC* signet ring cell carcinoma, *AJCC* American Joint Committee on Cancer

### Survival

**Fig. 2:**
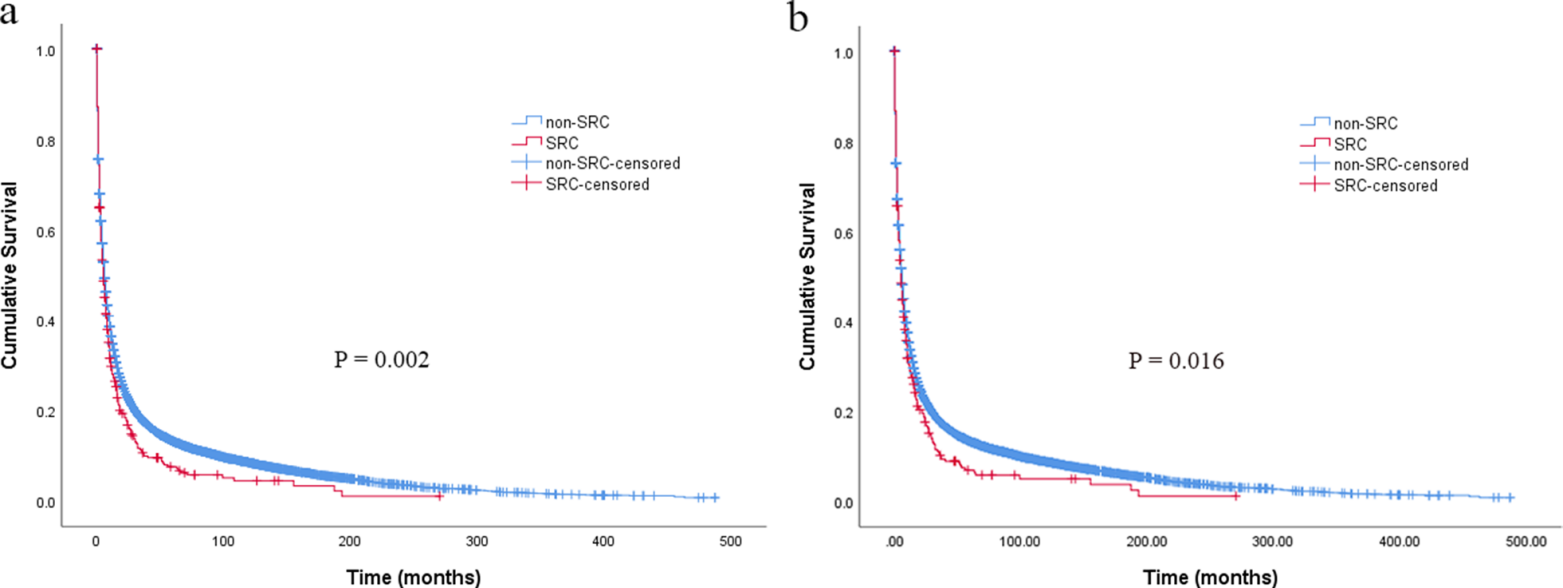
Overall survival (**a**) and cancer-specific survival (**b**) of patients with signet ring cell carcinoma and non-signet ring cell carcinoma

The median follow-up period was 5 (range 0–270) months for SRC group and 6 (range 0–487) months for non-SRC group. The 1-, 2- and 5-year OS was 28.1%, 16.8% and 7.2% for SRC *vs*. 34.9%, 23.1% and 13.2% for non-SRC, respectively (*P* = 0.002) (Fig. 2a). The 1-, 2- and 5-year CSS was 29.0%, 10.3% and 6.5% for SRC *vs*. 33.8%, 17.6% and 13.3% for non-SRC, respectively (*P* = 0.012) (Fig. 2b). In multivariable analysis, SRC was an independent determinant of OS (HR = 1.256, 95% CI 1.035–1.523, *P* = 0.021) and CSS (HR = 1.211, 95% CI 1.012–1.447, *P* = 0.036) (Table 2).

**Table 2:** Prognostic factors for survival

Characteristic	Overall survival				Cancer-specific survival			
	Univariate analysis		Multivariate a		Univariate Analysis		Multivariate analysis	
	HR (95% CI)	*P* Value	HR (95% CI)	*P* Value	HR (95% CI)	*P* Value	HR (95% CI)	*P* Value
*Age, years*								
≤ 60	Reference		Reference		Reference		Reference	
> 60	1.428 (1.384–1.473)	0.001	1.484 (1.383–1.594)	0.001	1.516 (1.459–1.575)	0.001	1.357 (1.266–1.456)	0.001
*Sex*								
Male	Reference		Reference		Reference		Reference	
Female	0.950 (0.922–0.980)	0.001	0.894 (0.833–0.960)	0.002	0.953 (0.923–0.984)	0.003	0.900 (0.845–0.959)	0.001
*Race*								
White	Reference		Reference		Reference		Reference	
Black	0.980 (0.935–1.027)	0.395	1.065 (0.965–1.175)	0.210	0.967 (0.920–1.016)	0.182	0.995 (0.913–1.085)	0.914
Other	0.847 (0.808–0.888)	< 0.001	0.907 (0.815–1.009)	0.073	0.862 (0.822–0.905)	< 0.001	0.882 (0.804–0.967)	0.008
*Clinical T-stage*								
T1–2	Reference				Reference			
T3–4	2.710 (2.584–2.841)	< 0.001			2.636 (2.504–2.774)	< 0.001		
*Lymph node metastasis*								
No	Reference				Reference			
Yes	1.425 (1.359–1.494)	< 0.001			1.406 (1.337–1.479)	< 0.001		
*Distant metastasis*								
No	Reference				Reference			
Yes	3.101 (2.965–3.244)	< 0.001			3.031 (2.889–3.180)	< 0.001		
*AJCC clinical stage*								
I–II	Reference		Reference		Reference		Reference	
III–IV	3.210 (3.066–3.361)	< 0.001	2.807 (2.519–3.041)	< 0.001	3.105 (2.956–3.261)	< 0.001	2.816 (2.622–3.023)	< 0.001
*Surgery*								
No	Reference		Reference		Reference		Reference	
Yes	0.370 (0.355–0.387)	< 0.001	0.575 (0.519–0.637)	< 0.001	0.345 (0.332–0.359)	< 0.001	0.525 (0.481–0.573)	< 0.001
*Radiation*								
No	Reference		Reference		Reference		Reference	
Yes	0.605 (0.578–0.634)	< 0.001	0.908 (0.820–1.006)	0.065	0.602 (0.573–0.632)	< 0.001	0.966 (0.882–1.057)	0.450
*Chemotherapy*								
No	Reference		Reference		Reference		Reference	
Yes	0.848 (0.821–0.875)	< 0.001	0.801 (0.738–0.869)	< 0.001	0.837 (0.810–0.865)	< 0.001	0.673 (0.627–0.722)	< 0.001
*Histologic grade*								
Well-moderate	Reference		Reference		Reference		Reference	
Poor-undifferentiated	1.838 (1.774–1.903)	< 0.001	1.632 (1.527–1.745)	< 0.001	1.826 (1.759–1.894)	< 0.001	1.686 (1.589–1.789)	< 0.001
*Histology*								
Non-SRC	Reference		Reference		Reference		Reference	
SRC	1.184 (1.063–1.320)	0.002	1.256 (1.035–1.523)	0.021	1.157 (1.027–1.304)	0.016	1.211 (1.012–1.447)	0.036
*Year of diagnosis*								
1998–2009	Reference		Reference		Reference		Reference	
2010–2016	0.796 (0.771–0.823)	< 0.001	0.912 (0.848–0.981)	0.013	0.800 (0.773–0.828)	< 0.001	0.884 (0.834–0.937)	< 0.001
*Marital status*								
Unmarried	Reference		Reference		Reference		Reference	
Married	0.819 (0.796–0.842)	< 0.001	0.865 (0.809–0.924)	< 0.001	1.249 (1.213–1.286)	< 0.001	1.221 (1.151–1.295)	< 0.001

*HR* hazard ratio, *CI* confidence interval, *AJCC* American Joint Committee on Cancer, *SRC* signet ring cell carcinoma

Table 3 summarizes the characteristics of the patients in the PSM analysis. There were no differences in baseline confounding variables between the two groups. After matching, SRC still had prognostic value for OS (HR = 1.341, 95% CI 1.006–1.687, *P* = 0.012) and CSS (HR = 1.625, 95% CI 1.162–2.273, *P* = 0.005). The 5-year OS in patients with SRC was 8.0% compared with 14.9% in patients with non-SRC. The 5-year CCS in patients with SRC was 8.5% compared with 13.4% in patients with non-SRC.

**Table 3:** Patient characteristics after propensity score matching

	SRC (n = 245)	Non-SRC (n = 245)	*P* Value
*Age, years*			
≤ 60	56 (22.9%)	49 (20.0%)	0.441
> 60	189 (77.1%)	196 (80.0%)	
*Sex*			
Male	59 (24.1%)	62 (25.3%)	0.753
Female	186 (75.9%)	183 (74.7%)	
*Race*			
White	194 (79.2%)	181 (73.9%)	0.097
Black	28 (11.4%)	29 (11.8%)	
Other	23 (9.4%)	35 (11.3%)	
*AJCC stage*			
I–II	110 (44.9%)	110 (44.9%)	1.000
III–IV	73 (29.8%)	73 (29.8%)	
Unknown	62 (25.3%)	62 (25.3%)	
*Histologic grade*			
Well-moderate	18 (7.3%)	18 (7.3%)	1.000
Poor-undifferentiated	227 (92.7%)	227 (92.7%)	
*Surgery*			
Yes	219 (89.4%)	220 (89.8%)	0.882
No	26 (10.6%)	25 (10.2%)	
*Radiotherapy*			
Yes	38 (15.5%)	30 (12.2%)	0.296
No	207 (84.5%)	215 (87.8%)	
*Chemotherapy*			
Yes	85 (34.7%)	91 (37.1%)	0.572
No	160 (65.3%)	154 (62.9%)	
*Year of diagnosis*			
1975–2009	142 (58.0%)	60 (24.5%)	< 0.001
2010–2016	103 (42.0%)	185 (75.5%)	
*Marital status*			
Married	127 (51.8%)	119 (48.6%)	0.471
Unmarried	118 (48.2%)	126 (51.4%)	

*SRC* signet ring cell carcinoma, *AJCC* American Joint Committee on Cancer

The effect of treatment types were further analysed. Of the 377 gallbladder SRC patients, 99 with undefined treatment information were excluded. In the remaining 278 patients, 153 (55%) received surgery alone, 14 (5%) received surgery in combination with radiotherapy, 79 (28.4%) received surgery in combination with chemotherapy, and 32 (11.5%) received chemotherapy alone. Comparison of OS between patients who underwent surgery and those who received chemotherapy alone showed that the long-term survival of patients who received surgery in combination with chemotherapy, but not with radiotherapy, were significantly better than those who received surgery or chemotherapy alone (Table 4).

**Table 4:** Prognosis of patient with signet ring cell carcinoma stratified by treatment

Variables	N	Age, years > 60	Male	AJCC III—IV stage	5-year OS (%)	HR (95% CI)	*P* value
*Whole group*	*278*						
CT alone	32	25 (78.1%)	4 (12.5%)	22 (68.8%)	0	Reference	
Surgery alone	153	122 (79.7%)	39 (25.5%)	37 (24.2%)	7.8	0.605 (0.403–0.909)	0.015
Surgery + RT	14	13 (92.9%)	6 (42.9%)	1 (7.1%)	0	0.478 (0.244–0.937)	0.032
Surgery + CT	79	50 (63.3%)	15 (19.0%)	27 (34.2%)	8.6	0.433 (0.279–0.671)	< 0.001
*Surgery group*	*246*						
Surgery alone	153	122 (79.7%)	39 (25.5%)	37 (24.2%)	7.8	Reference	
Surgery + RT	14	13 (92.9%)	6 (42.9%)	1 (7.1%)	0	0.802 (0.444–1.451)	0.467
Surgery + CT	79	50 (63.3%)	15 (19.0%)	27 (34.2%)	8.6	0.726 (0.538–0.980)	0.036

*OS* overall survival, *HR* hazard ratio, *CI* confidence interval, *CT* chemotherapy, *RT* radiotherapy,

*AJCC* American Joint Committee on Cancer

## Discussion

The clinicopathological characteristics and prognosis of patients with gallbladder SRC remain unclear, possibly because of its rarity. Current knowledge about gallbladder SRC is mainly extrapolated from anecdotal case reports, with limited statistical power [6–17]. It is therefore necessary to undertake an analysis on gallbladder SRC based on large databases such as SEER that can provide a more comprehensive and larger sample size cohort of patients. To the best of our knowledge, this is the first population-based analysis to describe the clinicopathological characteristics, prognosis and treatment strategies specific to gallbladder SRC.

In this large population-based study, 22,781 patients with gallbladder adenocarcinomas (SRC and non-SRC) were identified from the SEER database, of whom 1.7% patients were diagnosed with gallbladder SRC. The mean age of the SRC patients was 69.0 years in our cohort, similar to the mean age of 61.3 (range 22–86) years reported in the previous articles [6–17]. Contrary to the finding of male predilection for primary SRC in other sites, such as the pancreas and colon, our study showed that the male–female ratio was 0.30 for gallbladder SRC, presenting a female predilection [18–21]. This difference may be caused by the female-predilection nature of gallbladder carcinoma itself [22]. Among this cohort, we found that patients with gallbladder SRC were more significantly associated with older age, female gender, poor differentiation, advanced tumor stage, lymph node metastasis, distant metastasis, and advanced AJCC stage than those with non-SRC. When adjusting for other clinical and demographical features that were available, SRC was identified as an independent negative prognostic factor in patients with gallbladder adenocarcinomas. Although SRC exhibits dedifferentiated, highly malignant and aggressive properties, its mechanism remains unclear. Previous articles have reported that the abnormal activation of ErbB2/ErbB3 or loss of E-cadherin and MUC4 may deprive signet ring cells of the ability to maintain cell-to-cell contact, thereby promoting invasion and metastasis [23–26]. This mechanism may partly explain the high metastasis rate and poor prognosis of SRC, as derived from our analyses.

Given the poor prognosis of gallbladder SRC, it is necessary to find an optimal treatment strategy. Total tumor excision with adjuvant chemoradiotherapy is the mainstay of treatment for gallbladder adenocarcinomas at present [27, 28]. However, no standardized protocol and guideline for the treatment of gallbladder SRC are available at present because of the limited number of cases and studies. In the previous 12 cases reported, five patients underwent surgery with chemotherapy [6, 8, 10, 13, 17], three underwent surgery alone [7, 9, 12], one underwent surgery with chemoradiotherapy [14], two received no treatment [11, 15], and one had no detail information [16]. In our analysis, we found that patients who underwent surgery, with or without chemotherapy or radiotherapy, had better survival than those who received chemotherapy alone (Table 4). When compared with surgery alone, we found an interesting trend, showing that patients who underwent surgery with chemotherapy had significantly improved OS (*P* = 0.036), whereas no difference in OS was shown in patients who underwent surgery with radiotherapy (*P* = 0.467), suggesting that surgery with chemotherapy may be the optimal treatment for gallbladder SRC, which is consistent with the traditional management strategy of SRC in other sites [29–31]. As for adjuvant radiotherapy, no benefit was obtained in our study, and a similar result was also reported in a study involving 51 patients with stage II rectal SRC [32]. In addition, previous studies have reported that SRC histology seems associated with resistance to radiotherapy in patients with cervical and esophageal adenocarcinoma [33, 34]. Therefore, adjuvant radiotherapy is not recommended for routine treatment of SRC.

The present study represents the first and largest study on gallbladder SRC to date, but several limitations remain. Firstly, selection bias could not be ignored due to the retrospective nature of the study. In addition, some important information about therapies was not recorded in the SEER database, such as the radiation dosage and chemotherapy regimens. Meanwhile, some important variables associated with survival, including co-morbidities and the resection margin status, which would greatly impact survival, were also not accessible. Finally, we did not study the effect of radiotherapy alone on survival, for no patient in our cohort received radiotherapy alone. Despite these limitations, the results of this study can still provide clinicians with deeper insights into this rare tumor.

## Conclusion

SRC of the gallbladder has a worse prognosis than non-SRC, with poorer differentiation, and a more advanced stage. Surgery with chemotherapy is the main treatment strategy to improve survival, which supports the traditional management strategy of SRC. However, no survival advantage was obtained from adjuvant radiotherapy in the current study.

## Data Availability

The data that support the findings of this study were abstracted from an open database, the Surveillance, Epidemiology, and End Results (SEER) database (https://seer.cancer.gov).

## Abbreviations

SRC: Signet ring cell carcinoma
OS: Overall survival
HR: Hazard ratio
CI: Confidence interval
SEER: Surveillance, epidemiology, and end results
AJCC: American Joint Committee on Cancer
